# Modic changes in the cervical endplate of patients suffering from cervical spondylotic myelopathy

**DOI:** 10.1186/s13018-018-0805-2

**Published:** 2018-04-18

**Authors:** Pan Qiao, Tian-Tong Xu, Wen Zhang, Rong Tian

**Affiliations:** 1 Department of Spine Surgery, Tianjin Union Medical Center, 190 jieyuan Road, Hongqiao District, Tianjin, 300121 People’s Republic of China; 2 Department of Pediatrics, Tianjin Children’s Hospital, Tianjin, 300000 China

**Keywords:** Modic changes, Bone marrow edema, Cervical spondylotic myelopathy, Disc degeneration, Cervical endplate

## Abstract

**Background:**

The distribution and related factors of Modic changes (MC) in the lumbar spine has been evaluated. In the present study, the MC in the cervical endplate of patients with cervical spondylotic myelopathy (CSM) was investigated.

**Methods:**

A total of 6422 cervical endplates of 539 patients suffered from CSM (259 males and 280 females) with mean age of 46 ± 5.2 years. All patients underwent MRI scans and X-ray for evaluating the distribution of MC. The clinical information were recorded.

**Results:**

It was observed that 13.0% of patients and 2.4% of endplates showed MC. There were 3.7, 7.6, and 1.7% of cases diagnosed as types I, II, and III, respectively, suggesting MC were corrected with disc degeneration. The incidence rates of MC were 0, 0.3, 0.6, 0.9, 0.7, and 0.2%, respectively, in different intervertebral disc segments C_2–3_, C_3–4_, C_4–5_, C_5–6_, C_6–7_, and C_7_T_1_. Disc degeneration, segment, disease course, and age were statistically related to the MC. Patients over the age of 40 more easily suffered from MC.

**Conclusions:**

MCs manifested as type II mainly in patients with CSM. The incidence was highest in the C_5–6_ segment. Disc degeneration greatly contributed to the occurrence of MC.

## Background

Modic changes (MC) are signal intensity changes of vertebral endplates and subchondral bone as observed upon MRI signal changes. Abnormal signals in the adjacent vertebral endplate region were first reported by de Roos et al. [1] in 1987 in patients with degenerative disease using lumbar MRI. In 1988, Modic et al. [1, 2] systematically described the type, classification standard, and histologic changes in MRI signals. For now, many researchers have focused on the aspects of lumbar MC [3], and the changes and outcomes and their relationships with low back pain has been made clear [3–11], and the relations between MC and intervertebral disc degeneration or the motion segments have been confirmed. However, studies on MC in the cervical endplate, especially in the occurrence of cervical myelopathy, are still limited.

To date, the factors related to MC in the cervical endplate are not clear. The influence of the condition of the cervical curve representing the status of the cervical spine and the durations of symptoms reflecting the time from the symptoms’ beginning have not been investigated.

Cervical spondylotic myelopathy (CSM) is a chronic degenerative condition of the cervical spine that produces narrowing of the spinal canal and disruption of spinal cord function and accounts for about 10–15% of cervical spondylosis [12]. It mostly occurs in middle-aged and older patients, but the prevalence in younger ages has obviously increased in recent years. Our understanding of the disease has been increased since 1952, when Brain et al. [13] reported a large group of cervical spondylosis patients and divided them into myelopathic type and nerve root type.

With the progress in and application of modern medical imaging technology, especially MRI, MCs are easier to be found in patients with CSM. However, studies are still lacking regarding the distribution and related factors.

Therefore, in this study, we analyzed retrospectively the distribution and related factors regarding MC in the cervical endplate of cervical spinal cord cases and compare the findings with the other studies on MC in the cervical spine mentioned above as well as correlate with similarly designed studies conducted on the lumbar region.

## Methods

### General data

Five hundred thirty-nine patients with available cervical MRI scans and X-ray were chosen from January 2012 and September 2015 in our institution, who were diagnosed as CSM. A total of 259 men and 280 women ranging from 24 to 87 years (mean age, 46 ± 5.2) were statistically analyzed in this study.

All of the patients with chronic axial symptoms such as neck and shoulder pain as well as muscle cramps accompanied with neck stiffness and limited mobility chosen in this study were resulted from single-level or multilevel cervical disk degeneration confirmed by cervical MRI scans and X-ray.

The general exclusion criteria were as follows according to the imaging reports or noted on the MRI: (1) suffering from recent acute vertebral fractures such as Schmorl’s nodes, surgical fusions, spinal infections, or tumors; (2) patients with inflammatory spondyloarthropathy, hemodialysis spondyloarthropathy, or congenital or acquired block vertebrae; (3) undergoing radiotherapy; or (4) smokers.

Imaging characteristics and classification of MC were standardized based on the literature and agreed by consensus within the two readers. Before any studies began, a sample set of images were evaluated by two radiologists and held an in-person meeting to review and refine the standardized definitions. Because this was a retrospective study using data routinely collected, according to a waiver issued by the ethics committee, specific ethics approval for this study was not required. Each patient was assigned a number, and data for patient age, sex, presence or absence of MC, MC type, and the respective segmental level were collected.

We analyzed retrospectively the features of the distribution of MC of the cervical endplate with respect to age, durations of symptoms, spinal segment, and grade of intervertebral disc degeneration and firstly used the endplate instead of the motion segment as a statistical unit regarding the distribution of MC.

### Imageological examination

For the MR examination, we used an American GE Signa CV/i-type 1.5T magnetic resonance scanner (Fairfield City, CA, USA). Regarding T2WI, TR/TE was 3000 ms/100 ms; and for TlWI, TR/TE was 560 ms/12 ms. Coli was USCSl2, NEX was 2.00, matrix was 320 × 256, and FOV was 24. Thickness/layer was 3.0 mm/1.0 mm. The scanning software used was No Phase Wrap, Variable Bandwidth, Tailored RF pulses.

For the X-ray examination, we used a GE Advantx RFX type 90 X-ray machine (Fairfield City, CA, USA). The voltage was anteroposterior 75 kV/lateral 85 kV, at an electric current of 500 mA. We used an automatic exposure control system (Fairfield City, CA, USA).

### Image analysis

**Fig. 1:**
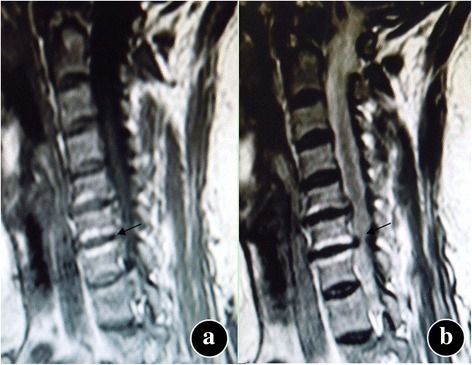
Modic type I changes. Hemispheric low signal intensity on T1-weighted (**a**) and high signal intensity on T2-weighted (**b**) images are noted on both sides of the endplate at the C6–7 level in the cervical spine

**Fig. 2:**
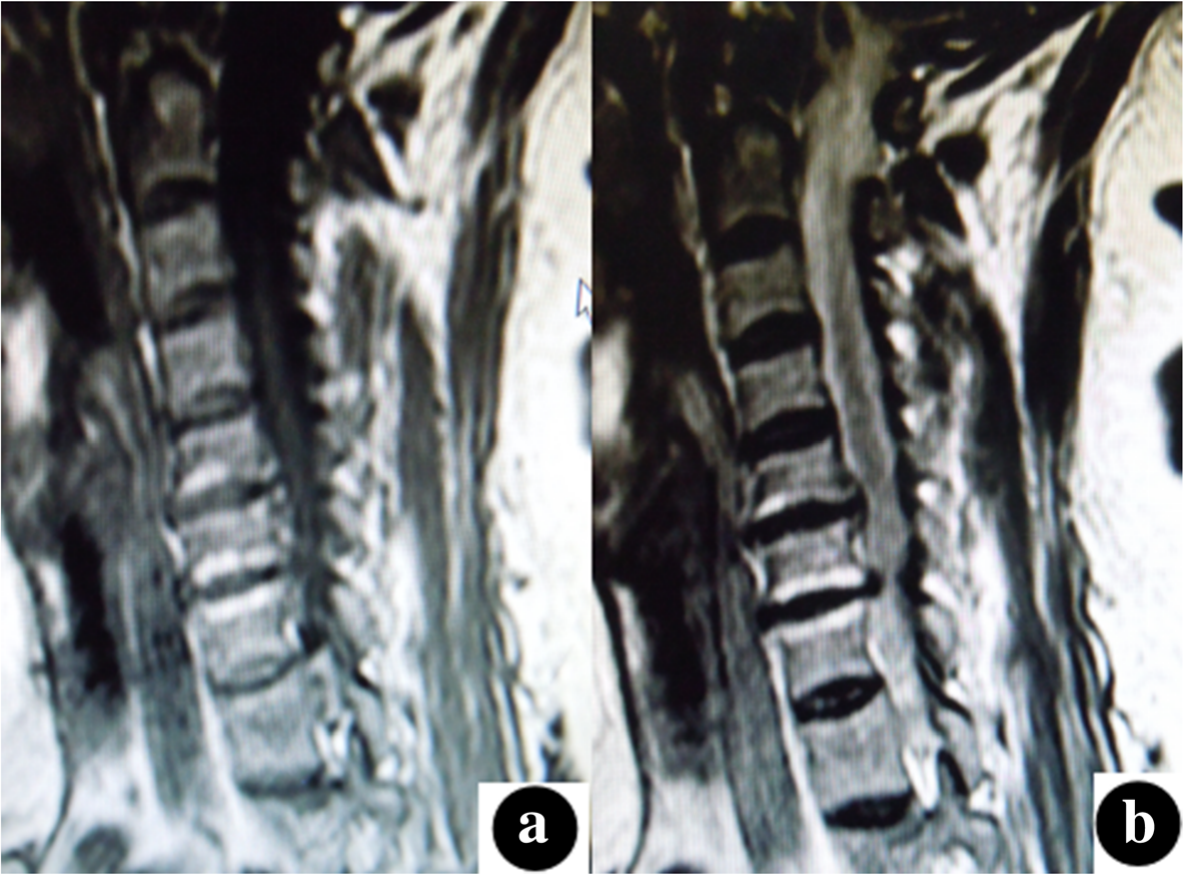
Modic type II changes. High signal intensity on T1-weighted (**a**) and T2-weighted (b) images are noted on both endplates at the C6–7 level in the cervical spine

**Fig. 3:**
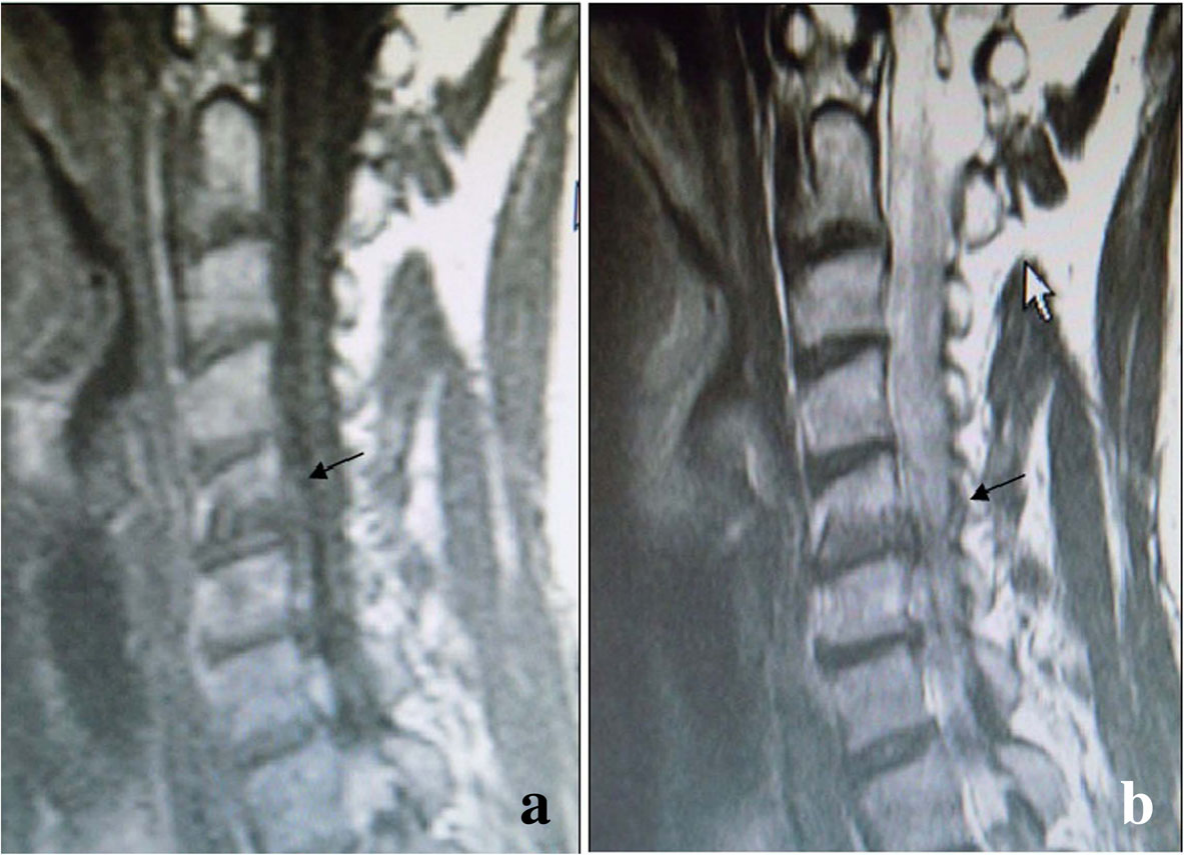
Modic type III changes. Hemispheric low signal intensity on T1-weighted (**a**) and T2-weighted (b) images is noted on the upper endplate at the C5–6 level in the cervical spine

The types of MC were according to MC standards as assessed by MRI [1, 2]: type 0: signal of the cervical endplate was normal, type I: hypointense signal on T1-weighted sequences and hyperintense signal on T2-weighted sequences, type II: hyperintense signal on T1 sequences and hyper- or isointense signal on T2 sequences, and type III: hypointense signal on T1 and T2 sequences (Figs. 1, 2, and 3). Pfirrmann grading standards [14] of intervertebral disc degeneration was shown in Table 1. Cervical curvature was measured by the C2–C7 Cobb’s angle. Cobb’s angle C2–7 was measured by formal Cobb methods that checked angle between the horizontal line of C2 lower end plate and the horizontal line of C7 lower end plate [15, 16]. In this study, the values of an angle below 0° were classified as recurvation and the values of 0° or above were classified as non-recurvation.

**Table 1:** Pfirrmann grading standards in MRI (T2WI) of intervertebral disc degenerations

Grading	Construction	Border of nucleus pulposus and fibrous rings	Signal	Disc height
I	Homogeneous, bright white	Distinct	High signal or equal to CSF	Normal
II	Heterogeneous, with or without horizontal belt	Distinct	High signal or equal to CSF	Normal
III	Heterogeneous, gray	Ambiguous	Medium signal	Normal to moderately reduced
IV	Heterogeneous, gray or black	Disappear	Medium or low signal	Normal to moderately reduced
V	Heterogeneous, black	Disappear	Low signal	Disc collapse

### Statistical methods

All data were collected and statistical analyzed by SPSS Version 19.0 (Chicago, IL, USA). Cohen’s kappa statistics was used to calculate intra- and interrater reliability. Comparisons with values of *P* < 0.05 were considered statistically significant. Results were presented as mean ± standard deviation.

## Results

### Distribution of Modic changes in all patients

The intraobserver agreement with the Modic classification was excellent (weighted kappa 0.86). The interobserver agreement was substantial (weighted kappa 0.73). Among 539 patients with 3211 motion segments or 6422 endplates, 70 (13.0%) patients and 88 (154 endplates or 2.4%) motion segments showed MC. Twenty (3.7%) cases and 27 (51 endplates or 0.8%) motion segments were type I; 41 (7.6%) cases and 43 (77 endplates or 1.2%) motion segments were type II; and 9 (1.7%) cases and 18 (26 endplates or 0.4%) motion segments were type III. According to the occurrence of MC in different intervertebral disc segments, 0 (0%) lesions involved C2–3, 9 (0.3%) involved C3–4, 20 (0.6%) involved C4–5, 29 (0.9%) involved C5–6, 23 (0.7%) involved C6–7, and 7 (0.2%) involved C7T1 (Table 2).

**Table 2:** Distribution of MC in different intervertebral disc segments

MC	Intervertebral disc segments						Total
	C_2-3_	C_3-4_	C_4-5_	C_5-6_	C_6-7_	C_7_-T_1_	
I	0	4	6	9	6	2	27
II	0	5	10	12	13	3	43
III	0	0	4	8	4	2	18
Total	0	9	20	29	23	7	88

### The distribution of Modic changes with different degrees of disc degeneration

The morbidity of MC using different classifications of disc degeneration was shown in Table 3. As can be seen, the morbidities among the five groups were significant differences (*χ*^*2*^ = 223.137, *P =* 0.000).

**Table 3:** Distribution of MC in different Cobb angle groups

MC	Number of cases		Total
	Recurvation group	Non-recurvation group	
Normal	149	302	451
MCI, II or III	52	36	88
Total	201	338	539

### The distribution of Modic changes at different degrees of cobb angles

Table 4 shows the morbidity of MC in groups with different Cobb angles, representing a statistically significant difference (*χ*^*2*^ = 7.659, *P* = 0.006).

**Table 4:** Distribution of MC in both genders

Genders	Number of cases		Total
	Female	Male	
Normal	234	231	465
MC	31	43	74
Total	265	274	539

### The distribution of Modic changes in different sex groups

The distribution of MC in both sexes was shown in Table 5. However, there was no significant difference between female and male groups (*χ*^*2*^ = 2.569, *P* = 0.109).

**Table 5:** Distribution of MC in different age groups

Age	Number of cases							
	< 29	30–39	40–49	50–59	60–69	70–79	80–89	Total
Normal	8	85	104	123	82	43	16	461
MC	0	6	14	25	23	5	5	78
Total	8	91	118	148	105	48	21	539

### The distribution of Modic changes in different age groups

Table 6 shows the distribution of MC in different age groups. The younger than 40 years and the older group represented a statistical significant difference (*χ*^*2*^ = 57.437, *P* = 0.000).

**Table 6:** Distribution of MC in different durations of symptoms

MC	Number of cases						
	< 6 m	6–12 m	12–18 m	18–24 m	24–30 m	30–36 m	Total
Normal	95	72	105	94	41	65	472
MC	2	4	19	8	10	24	67
Total	97	76	126	102	51	89	539

“durations of symptoms” means a duration from the firstly appearing of the symptoms to the treatments in this study

### The distribution of Modic changes in different durations of symptoms

The distribution of MC in different durations of symptoms was shown in Table 7, representing a statistically significant difference between the groups with duration shorter than 18 months and the longer (*χ*^*2*^ = 23.438, P = 0.000).

### Overall correlation analysis

The above results suggest that the degree of disc degeneration, Cobb angle, age, disc segment, and durations of symptoms are all significantly correlated with MC. The regression equation was obtained using a binary logistic stepwise regression test as follows: *Y* = − 14.314 + 4.037*D* ± 0.784*C* + 0.310 *L* + 0.203 *T* + 0.162*A* (where *Y* denotes MC; *A*, age; *T*, durations of symptom; *L*, disc segment; *D*, the degree of disc degeneration; and *C*, Cobb angle group; *P* = 0.001, *EXP*: *D* = 23.038, *C* = 1.893, *L* = 1.307, *T* = 1.187, and *A* = 1.164). This calculation showed that the most relevant correlation was between disc degeneration and MC in cases of CSM; and this was followed by the Cobb angle grouping, intervertebral segment, durations of symptoms, and lastly age.

## Discussion

This article first used the endplate instead of the motion segment as the statistical unit regarding the distribution of MC. It is also describing for the first time the distribution of MC in the cervical endplate of patients suffering from CSM and the relevant correlation between durations of symptom and MC.

The present study demonstrated that 71 cases and 154 endplates showed MC, and the total prevalence was 13.2%. A previous prospective study conducted in the cervical spine with 497 originally asymptomatic healthy volunteers at baseline [17] showed the prevalence of all MC types was 4.5% of subjects, and 223 at follow-up [18] showed an increase to 13.9%. Thus, it can be seen that the prevalence of MC in the cervical spine was affected by the sample size. Jensen et al. [3] performed a meta-analysis of MC in the lumbar spine and found a difference in the incidence between different races, with Europeans exhibiting a higher prevalence of MC in the lumbar spine compared with Asians. Studies on cervical MC are very limited and lack sufficient statistical power due to small sample sizes. Whether such differences are related to regional and ethnic demographics requires further study.

The present study shows that the most common type of cervical MC is type II, with a rate of 1.2% (77 endplates) which might be for the reason that the enrollments were patients suffering from severe CSM with long course of spinal segmental degeneration. Two previous studies conducted in the cervical spine observed the same results [19, 20]. However, Peterson et al. [21] in Canada found that the most common type was type I. These authors argue that MC are associated with segmental instability, while the degree of activity of the cervical vertebrae is larger than in the lumbar spine. Therefore, further research with a wider age range of patients is needed. In addition, pay more attention to investigate the difference of original mechanism between types I and II are also necessary.

Our study shows that the most common areas for MC in the cervical spine are C5–6, and the prevalence is 1.0%. The similar outcomes have been reported. The C5–6 segments which are the concentrated stress site have the largest activity and range of motion among the cervical vertebrae and produce intervertebral disc degeneration [19–21]. However, it is still unclear as to which mechanism(s) affects endplate MC.

We showed that cervical MC are related to the degree of intervertebral disc degeneration, and that there is a higher prevalence over Pfirrmann grade III of degenerative discs and that its prevalence increases with the increase in the degree of disc degeneration. A consensus has been reached in this respect [18, 22]. The present study also suggests a very important correlation between MC and the degree of intervertebral disc degeneration, without a causal relationship. However, we cannot currently prove the evolution in types of MC due to the lack of long-term follow-up.

This study shows that the cervical MC are more common in recurvation group. It can be explained by cervical curvature reflecting its status and the recurvation reducing vertical shock absorption in a flattened spine. This would increase the risk of endplate damage during incidents such as a fall on the buttocks. Wu et al. have shown a higher prevalence of MC in patients with degenerative lumbar scoliosis [23], but this frontal-plane spinal curvature is primarily structural and cannot be equated with sagittal plane curvature which is more functional than structural.

The prevalence of cervical MC is not significantly different between sexes. Meanwhile, the same outcome was identified by Peterson et al. [21]. Conversely, the lumbar changes are correlated with sex, hormones, and profession [24, 25]. The reasons for this may be as follows. On the one hand, cervical vertebral bodies and intervertebral discs are more sensitive to overloading leading to reflective protection.

Therefore, there is no significant diffidence of cervical vertebra in different sexes, leading to no correlation between the prevalence of cervical MC and sexes.

This study shows that the cervical MC tend to occur in patients over 40 years of age. A previous prospective follow-up MRI study also reported age (≥ 40 years) was significantly associated with MC [18]. Furthermore, another study confirmed the prevalence of cervical MC increased with age [22].

This study shows that the cervical MC tend to occur in patients with durations of symptom above 18 months. Reasons may be that the degree of intervertebral disc degeneration becomes more severe as durations increases. The conversion and natural history of MC may be another important factor. Mann et al. [26] reported that no conversion from type II to type I or reverting to a normal image was observed in the cervical spine, in complete contrast to MC in lumbar spine.

This study shows that the degree of disc degeneration, Cobb angle, disc segment, durations of symptoms, and age are factors that correlate with MC. Previous researches [18, 22] suggesting that MC in the cervical endplate are associated with cervical intervertebral disc degeneration, which is likely to be one of the late manifestations of intervertebral disc degeneration. However, a strong statistical correlation between disc degeneration and endplate MC is still lacking enough support. Our evidence suggests that disc degeneration and MC of the endplate have a strong correlation. In each type of cervical spondylosis, cervical curvature change is one of the most common imaging manifestations and also an early sign of cervical spine instability. Attenuation of cervical anteflexion is a sign of the body’s protective response after cervical vertebra and intervertebral disc degeneration. However, the mechanism(s) that influence modifications in cervical curvature and endplate MC is arcane and requires further future study.

The durations of symptoms mainly including numbness and clumsiness of the limbs, numbness, inability to walk, “hit the cotton flu,” strangalesthesia of the thorax and abdominal regions, as well as severe bowel and bladder dysfunction, and age are also important factors in endplate MC. Age over 40 years predicts endplate MC and is also a predictor of cervical degeneration and disc degeneration. And as the durations of symptoms advances, cervical spondylosis of the myelopathic type becomes more aggravated [12], and degeneration commences gradually. This phenomenon is also confirmed by the close correlation among endplate MC and disc and cervical degeneration.

MC of the cervical endplate occur primarily in the C5–6 disc with type II predominating and type III least frequent. The prevalence of cervical MC is not significantly different between sexes. MC occur mainly after the age of 40 and are correlated with disc degeneration, disc level condition of the cervical curve, durations of symptoms and age; in addition, disc degeneration plays an important role in the occurrence of MC. Nevertheless, this study had several limitations: Firstly, the correlation of MC and symptoms or the severity of stenosis is not discussed. Secondly, our study seems to be a cross-sectional analysis, we should focus on the significance of MC in clinic treatment. Thirdly, we should next analyze the correlation of MC and symptomatology.

## Conclusion

In summary, our study reveals that Modic changes are distributed mainly over the age of 40 and are correlated with disc degeneration, disc level condition of cervical curve, course of disease and ages.

## Abbreviations

CSM: Cervical spondylotic myelopathy
MC: Modic changes
